# Evidence of Clinical Efficacy and Pharmacological Mechanisms of Resveratrol in the Treatment of Alzheimer’s Disease

**DOI:** 10.2174/0115672050272577231120060909

**Published:** 2023-12-30

**Authors:** Sian Jin, Xuefeng Guan, Dongyu Min

**Affiliations:** 1 Liaoning University of Traditional Chinese Medicine, Shenyang, 110000, China;; 2 Shenyang Pharmaceutical University, Shenyang, 110000, China;; 3 Affiliated Hospital of Liaoning University of Traditional Chinese Medicine, Shenyang, 110000, China

**Keywords:** Resveratrol, Alzheimer’s disease, meta-analysis, network pharmacology, PI3K signaling pathway, AD patients

## Abstract

**Background:**

To evaluate the efficacy and pharmacological mechanisms of resveratrol in Alzheimer’s disease (AD) patients.

**Methods:**

We conducted a thorough exploration of existing randomized controlled trials concerning the treatment of Alzheimer's disease patients using resveratrol, utilizing accessible open databases. Quantitative variables were represented as a standardized mean difference (SMD), accompanied by a 95% confidence interval (CI). Additionally, we examined the potential targets and plausible pathways associated with the impact of resveratrol on Alzheimer's disease using network pharmacology techniques.

**Results:**

Our meta-analysis comprised five trials involving 271 AD patients, of whom 139 received resveratrol treatment and 132 received placebo treatment. Compared with placebo therapy, resveratrol treatment resulted in a significant improvement in Alzheimer’s Disease Cooperative Study—Activities of Daily Living (ADAS-ADL) scores (SMD=0.51; 95% CI, 0.24 to 0.78) and cerebrospinal fluid (CSF) Aβ40 (SMD=0.84; 95% CI, 0.21 to 1.47) and plasma Aβ40 levels (SMD=0.43; 95% CI, 0.07 to 0.79). However, the improvement in the resveratrol-treated group compared with the placebo treatment group on the Mini-Mental State Examination (MMSE) score, CSF Aβ42 and plasma Aβ42 levels, and brain volume was not significant. There were no noteworthy statistical variances in the occurrence of adverse effects noted between the two groups. The outcomes of network pharmacology divulged that the principal enriched interaction pathway between resveratrol and Alzheimer's disease is primarily concentrated within the PI3K signaling pathways. Resveratrol's potential key targets for the treatment of AD include MAKP1, HRAS, EGFR, and MAPK2K1.

**Conclusion:**

While having a high safety profile, resveratrol has efficacy in AD patients to a certain extent, and more data are required to validate the efficacy of resveratrol for the treatment of AD in the future. Suppression of the PI3K signaling pathways could hold significant importance in the treatment of AD patients using resveratrol.

## 1 INTRODUCTION

Alzheimer’s disease (AD) is a progressive neurodegenerative disorder that affects millions of people worldwide, with an estimated 50 million cases globally [1]. This number is expected to triple by the year 2050, posing significant challenges for healthcare systems worldwide [2]. The disease is characterized by the accumulation of amyloid-beta (Aβ) plaques and neurofibrillary tangles, leading to cognitive decline and functional impairment [3]. Whereas current treatments can help manage symptoms of AD, there is an urgent need for more effective therapies to slow or halt disease progression.

Resveratrol a natural polyphenolic compound found in many plant species, including grapes, raspberries, peanuts, blueberries, mulberries, and knotweed [4-8]. Resveratrol exhibits strong anti-inflammatory characteristics owing to its ability to modulate small molecules in multiple signaling pathways to suppress the secretion of inflammatory factors [9, 10]. Therefore, resveratrol can have a positive impact on both the prevention and treatment of chronic inflammatory diseases, such as obesity, neurodegeneration, diabetes, cardiovascular diseases, and cancers, among other conditions [11-14]. Furthermore, due to its antioxidant, anti-inflammatory, and neuroprotective properties, resveratrol has gained significant interest as a potential therapeutic agent for AD [15, 16]. Numerous preclinical studies have demonstrated the benefits of resveratrol in mitigating various aspects of Alzheimer’s pathophysiology, including reduced Aβ aggregation, improved cognitive function, and enhanced synaptic plasticity [17, 18]. As such, resveratrol is a promising treatment option that warrants further investigation through rigorous clinical trials.

In this study, we aim to investigate the clinical efficacy and pharmacological mechanisms of resveratrol in the treatment of AD using a meta-analysis and network pharmacology approach. By gathering and analyzing data from relevant studies, our objective is to offer a comprehensive comprehension of the potential therapeutic advantages of resveratrol in the treatment of AD. Our ultimate goal is to provide valuable insights into the development of effective therapeutic interventions for AD.

## 2 MATERIALS AND METHODS

### 2.1 Meta-analysis

#### 2.1.1 Preparation of Randomized Controlled Study

In this meta-analysis, we adhered to the preferred reporting items for systematic reviews and meta-analyses (PRISMA) guidelines [19]. To extract relevant literature, we meticulously searched multiple renowned databases, including the PubMed Database, Embase, Web of Science, and Cochrane Library. Furthermore, we meticulously examined data sourced from reputable registries. We employed the following search terms: “Resveratrol,” “Alzheimer's Disease,” and “randomized trial,” without imposing any limitations on subheadings or language. Additionally, we examined the bibliographies of all the papers included in the study. We conducted a thorough manual review of literature not indexed in the aforementioned electronic databases but deemed potentially relevant.

#### 2.1.2 Criteria for Study Inclusion and Exclusion

The following criteria were used for inclusion: 1) Only research population with an AD diagnosis in line with applicable guidelines [20] were considered. 2) In the randomization assignment, patients were assigned to either the experimental (resveratrol or resveratrol supplements) group or the control (placebo) group. 3) Each study group comprised a minimum of 10 cases. 4) No constraints were placed on the duration of follow-up. 5) The outcomes were evaluated by quantitative cognitive assessment tools to enable the statistical analysis of outcomes.

The following criteria were used for exclusion: 1) Studies that were not randomized or were not conducted in a blinded manner. 2) Patients who were included in the study without a clear and confirmed diagnosis. 3) A comparison of various medications was conducted. 4) Outcomes of interest were not reported.

#### 2.1.3 Tools for Assessing Cognitive Function in Patients with AD

The following tools were used to evaluate the cognitive capacity of patients with AD. 1) The Alzheimer’s Disease Assessment Scale-Cognitive Subscale (ADAS-cog) [21], a widely accepted instrument for testing the effectiveness of interventions, is used to observe variations in behavior and mood and includes 11 items that require both participant-completed and observer-based examinations. Higher scores indicate poorer performance. 2) The Alzheimer’s Disease Cooperative Study-Activities of Daily Living (ADCS-ADL) scale [22], which comprises 23 items, was developed for use among patients with AD, and assigns a cumulative rating between 0 and 78, with a lower score indicating increased severity. 3) The Mini-Mental State Examination (MMSE) [23], a 30-point test of cognitive functions that evaluates the capacity for attention and orientation, along with the ability to recall, calculate, communicate, and draw. The total score is employed to infer the presence of cognitive deterioration, with lower scores signifying greater severity.

#### 2.1.4 Evaluation of Biomarkers Associated with Neuroinflammation in AD

Neuroinflammation may lead to cognitive impairment and play an important role in AD progression [24]. The neuroprotective mechanism of resveratrol may involve the reduction of neuroinflammation by reducing the accumulation and toxicity of Aβ protein in the brains of AD patients [25, 26]. Therefore, in order to test the anti-inflammatory effect of resveratrol in AD and also to understand the penetration of the drug into the blood-brain barrier, we measured the cerebrospinal fluid and plasma levels of two classical AD biomarkers (Aβ40 and Aβ42), respectively. The higher the level of Aβ protein after treatment (meaning that Aβ is stabilized and less likely to aggregate), the stronger the neurological anti-inflammatory effect of resveratrol, which may also be an intermediate process and potential mechanism for the improvement of cognitive impairment in AD patients [27, 28].

#### 2.1.5 Literature Screening and Data Extraction

Two researchers independently scrutinized papers, extracted data, and performed cross-checks. In cases of disagreement, a third party intervened. With the aid of EndNote software, we excluded duplicates and conducted preliminary screening by scrutinizing article titles and abstracts to discern which articles were suitable for absolute reading. Ultimately, utilizing an Excel data extraction sheet, they collected details, including the primary author, publication year, sample size, intervention methods, and outcome measures.

#### 2.1.6 Quality Assessment

Using the Cochrane Risk of Bias apparatus, we evaluated the risk of bias (ROB) present in the selected studies [29]. A total of seven ROB domains were assessed for each study, which were categorized as having high, low, or unclear ROB. The seven domains evaluated were “random sequence generation,” “allocation concealment,” “blinding of participants and personnel,” “blinding of outcome assessment,” “incomplete outcome data,” “selective reporting,” and “other bias”.

#### 2.1.7 Statistical Analysis

Data was synthesized using R software version 4.1.2 Continuous variables were represented as standardized mean differences (SMDs) with a 95% confidence interval (CI) [30]. To evaluate heterogeneity, we employed chi-square and I2 tests [31]. Where non-heterogeneous results (I2<50%) were observed, fixed effects models were utilized, and in the case of heterogeneous results (I2 ˃50%), random effects models were employed [32]. We defined statistical significance using a two-tailed test with *p*<0.05.

### 2.2 Network Pharmacology

#### 2.2.1 Forecasting Potential Targets of Resveratrol

We procured the 2D structure of resveratrol from PubChem [33] and utilized PharmMapper [34] to project the drug’s potential targets. To standardize gene information, all target names were inputted into UniProt [35] sites and restricted to Homo sapiens species.

#### 2.2.2 Target Screening for AD

The target genes related to AD were discovered using the keyword “Alzheimer's Disease” in the GeneCards [36] and DisGeNET [37] databases.

#### 2.2.3 Building of Protein-protein Interaction Network

To identify the potential interaction targets of resveratrol for treating AD, we utilized the online drawing tool Interactive Venn to create a Venn diagram. The intersection area of the Venn diagram contained the common targets of AD and resveratrol. The shared targets were inputted into the STRING 11.0 platform [38], where the protein-protein interaction network was constructed utilizing the STRING database and the Network Analyzer plugin of Cytoscape [39]. In the network, nodes with a higher degree indicate a stronger interaction. We conducted a screening of nodes with the highest degrees, which are thought to have a pivotal role in the network, grounded in their topological attributes.

#### 2.2.4 Enrichment Analysis

Gene ontology (GO) [40] enrichment and Kyoto Encyclopedia of Genes and Genomes (KEGG) [41] pathway enrichment analysis was executed using the Metascape [42] database to investigate the biological functions and potential mechanisms associated with the potential targets.

#### 2.2.5 Establishment of the Component–target–pathway Network

We used Cytoscape to create an integrated component–target–pathway network. Further, we assessed the network's topology parameters using the built-in Network Analyzer tool in Cytoscape. This analysis enhanced our comprehension of the interplay between protein targets, components, and the associated pathways. The flow chart for meta-analysis and network pharmacology is shown in Fig. (**1**).

## 3 RESULTS

### 3.1 Meta-analysis

#### 3.1.1 Features of included Studies

**Fig. (2):**
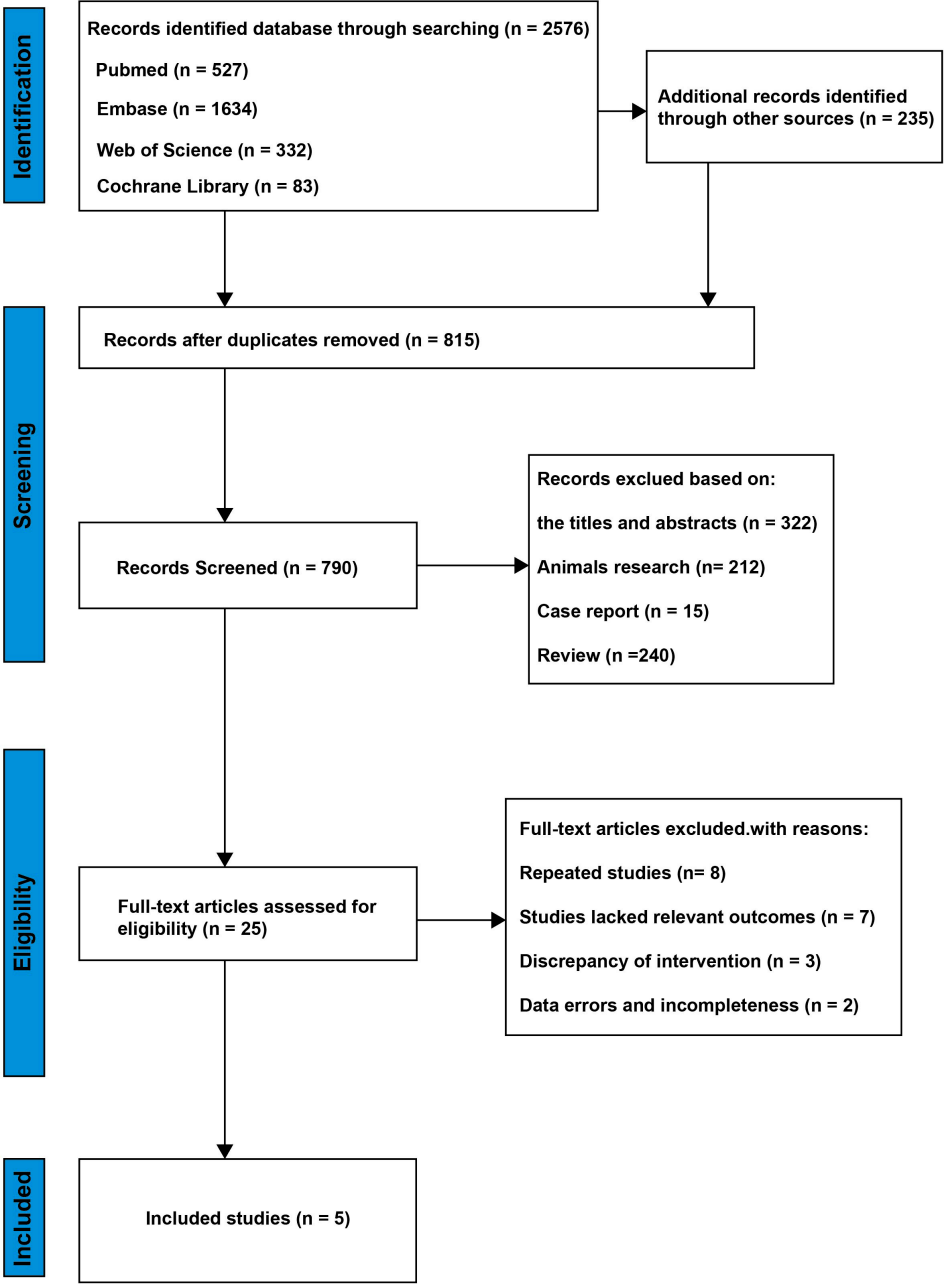
Flow chart of the systematic search process.

**Fig. (3):**
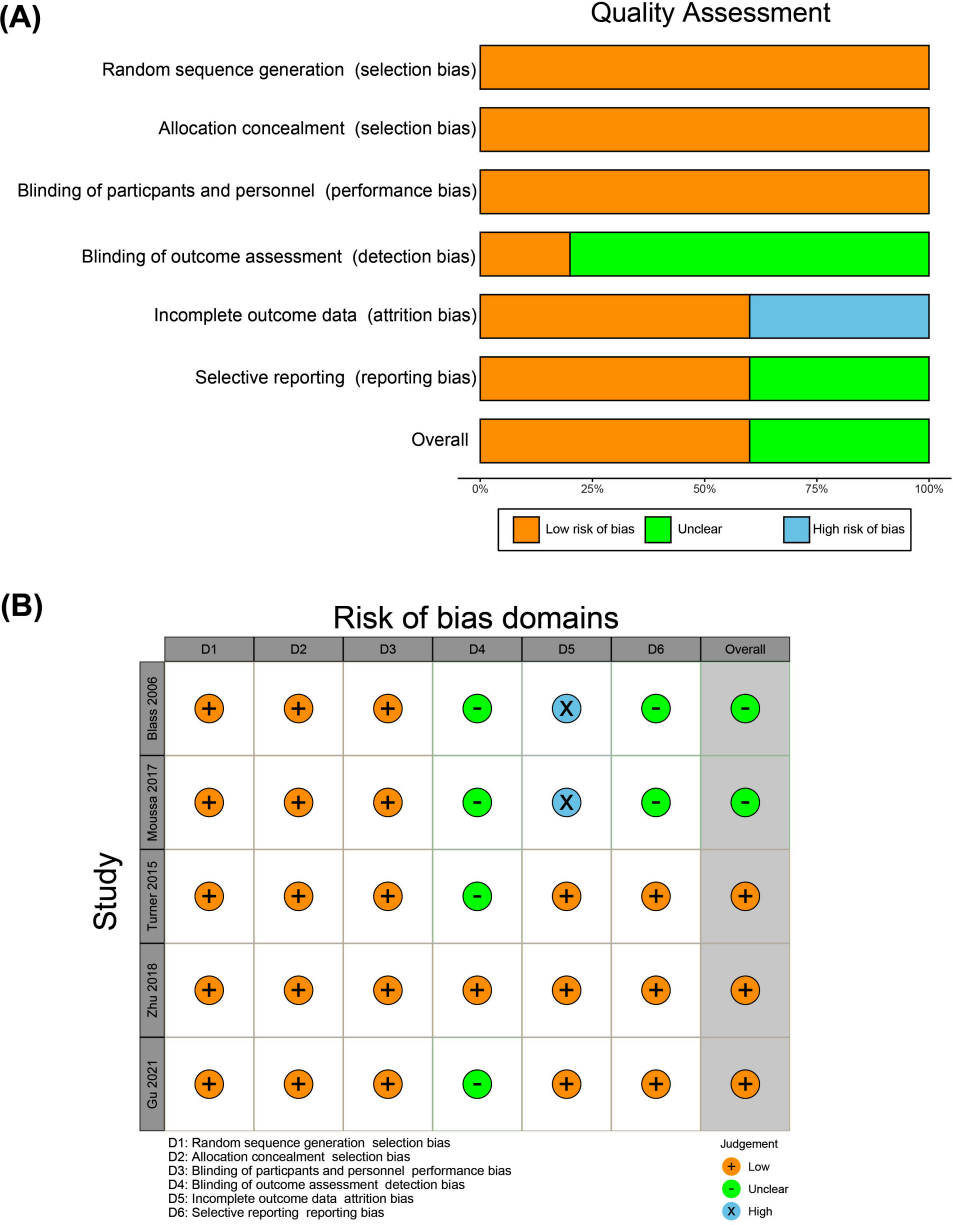
Quality assessment results. (**A**) Quality Assessment. (**B**) Risk of bias domains.

We initially screened 2,811 articles to identify potential candidates for inclusion. After a thorough stratification process, only five articles were deemed suitable [43-47]. Details of the literature selection process are illustrated in (Fig. **2**). The 271 patients included in the study were divided into two groups: experimental (n=139) and placebo-treated (n=132). The basic characteristics of the included articles are summarized in Table **1**. Notably, two of the studies used low doses of resveratrol (5 mg/day), while the other three used larger doses (500 mg/day). In addition, the quality of the literature for the five included studies is evaluated in (Fig. **3**).

#### 3.1.2 Cognitive Functions

**Fig. (4):**
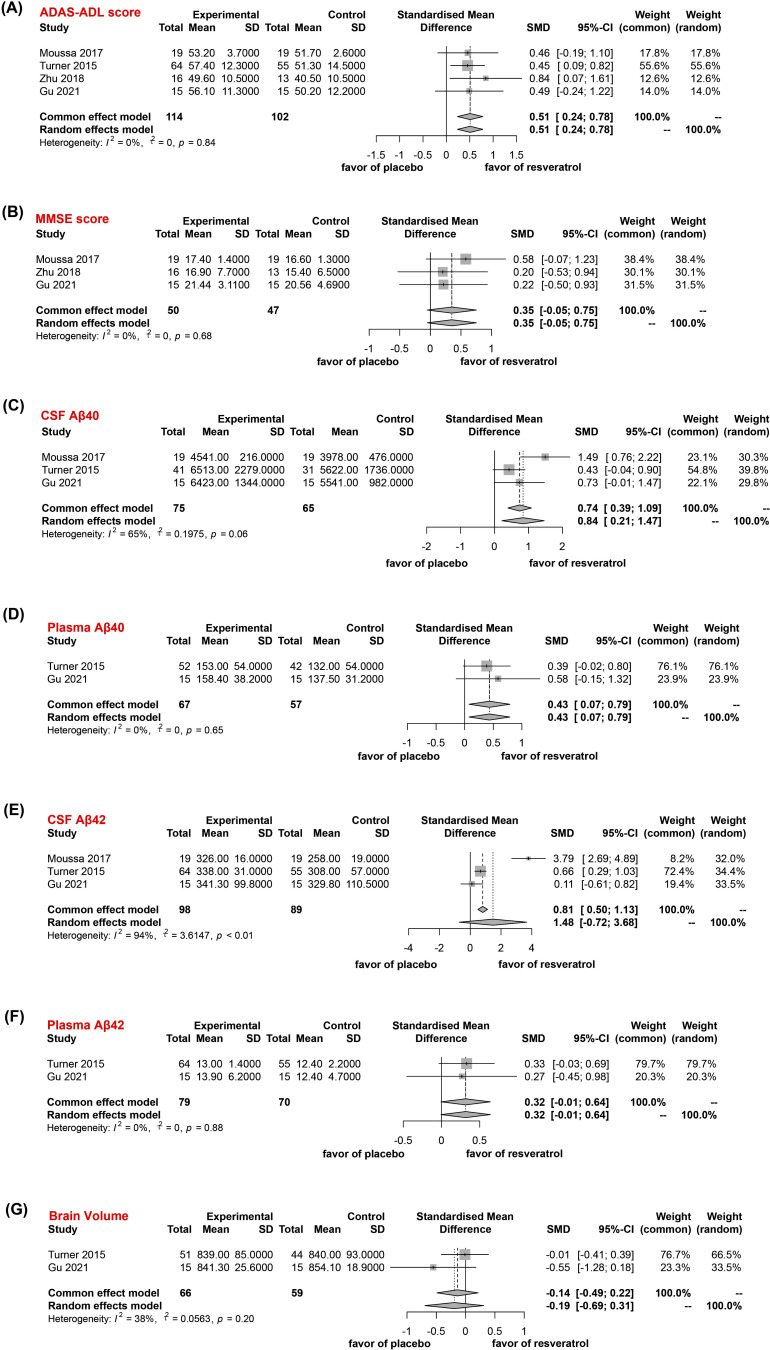
Meta analysis of the efficacy of resveratrol versus placebo in the treatment of AD patients. (**A**) ADAS-ADL score. (**B**) MMSE score. (**C**) CSF Aβ40. (**D**) Plasma Aβ40. (**E**) CSF Aβ42. (**F**) Plasma Aβ42. (**G**) Brain Volume.

Four studies reported ADAS-ADL scores for patients with AD after 52 weeks of treatment with resveratrol or placebo, and three studies reported MMSE scores. A significant improvement in ADAS-ADL scores was found in the resveratrol-treated group compared with the placebo-treated group (SMD=0.51; 95% CI, 0.24 to 0.78; I2=0; Fig. **4A**). Nevertheless, the MMSE score for the resveratrol-treated group did not exhibit a statistically significant difference compared to the placebo-treated group despite the observed improvement (SMD=0.35; 95% CI, -0.05 to 0.75; I2=0; Fig. **4B**). To reduce the bias in results due to dose differences, we excluded the resveratrol treatment results of Zhu *et al.* using a low dose (5 mg/day). We recalculated the outcome for the group using the 500 mg/day dose. We noted that the resveratrol-treated group still had a substantial improvement in ASAD-ADL scores compared with the placebo group (SMD=0.46; 95% CI, 0.17 to 0.75; I2=0; Fig. **S1A**), whereas the difference in MMSE scores between the two groups remained statistically indistinguishable (SMD=0.42; 95% CI, -0.07 to 0.90; I2= 0; Fig. **S1B**). In addition, the ADAS-cog score could not be analyzed due to an insufficient number of studies, including the data.

#### 3.1.3 Cerebrospinal Fluid and Plasma Markers

Three studies reported CSF Aβ40 and Aβ42 levels, and two studies reported plasma Aβ40 and Aβ42 levels in patients with AD after 52 weeks of treatment with resveratrol or placebo. The results showed that CSF Aβ40 (SMD=0.84; 95% CI, 0.21 to 1.47; I2=65%; Fig. **4C**) and plasma Aβ40 levels (SMD=0.43; 95% CI, 0.07 to 0.79; I2=0; Fig. **4D**) were significantly improved in the resveratrol-treated group compared with those in the placebo-treated group. Although the CSF Aβ42 (SMD=1.48; 95% CI, -0.72 to 3.68; I2=94%; Fig. **4E**) and plasma Aβ42 levels (SMD=0.32; 95% CI, -0.01 to 0.64; I2=0; Fig. **4F**) for the resveratrol-treated group were improved compared with those for the placebo-treated group, the results were not statistically significant.

#### 3.1.4 Brain Volume

Two studies reported brain volume in AD patients after 52 weeks of treatment with resveratrol or placebo. Although the brain volume in the resveratrol-treated group was less than that in the placebo-treated group, the result was not statistically significant (SMD=-0.14; 95% CI, -0.49 to 0.22; I2=38%; Fig. **4G**).

#### 3.1.5 Safety and Tolerability

Fig. (**5**) depicts a comparison of safety and tolerability in the two groups. No significant statistical differences in the incidence of adverse effects were observed between the two groups, with the exception of a lower rate of benign, malignant, and unspecified neoplasms observed in the resveratrol-treated group than in the placebo-treated group (RR=0.18; 95% CI, 0.03 to 0.95; I2=0). Furthermore, more than 20% of the adverse events in the resveratrol-treated group included Nervous system disorders (37%), Psychiatric disorders (38%), Infections and infestations, Gastrointestinal disorders (42%), Injury, poisoning, or procedural complications (29%), and Musculoskeletal and connective tissue disorders (20%).

### 3.2 Network Pharmacology

#### 3.2.1 Potential Targets of Resveratrol and AD

**Fig. (6):**
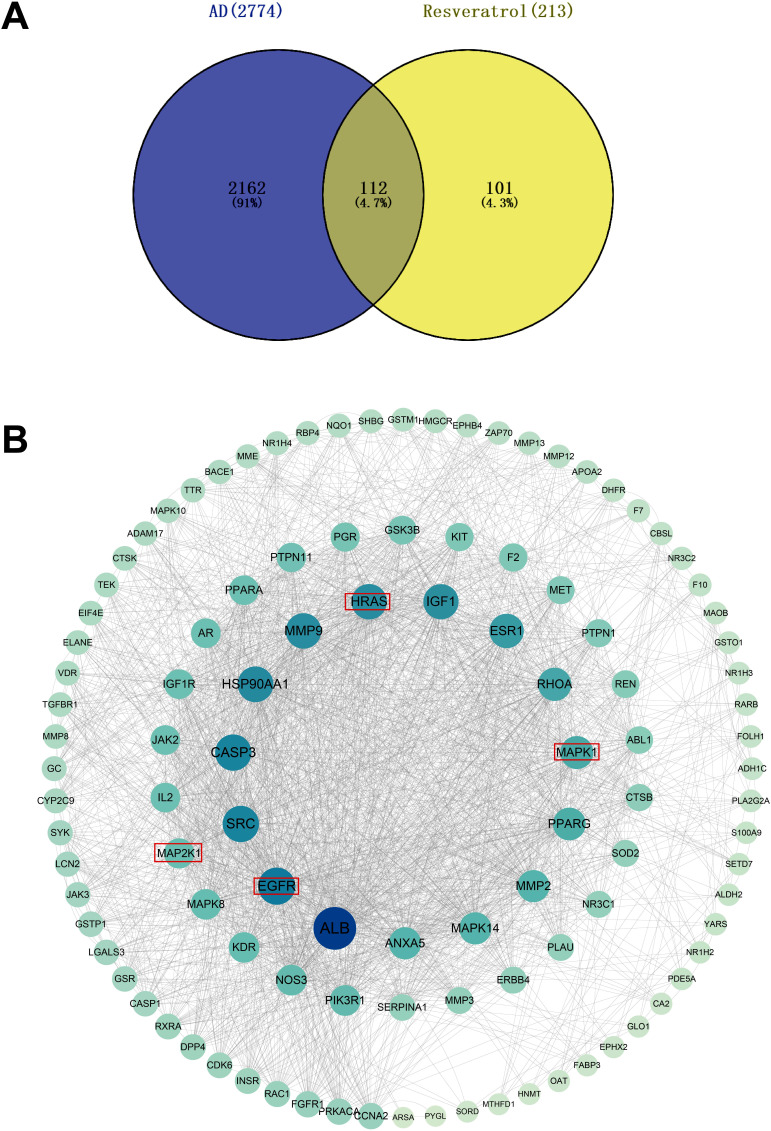
Venn diagram and PPI work. (**A**) Venn diagram. The blue section indicates AD-related targets, and the yellow section indicates resveratrol-related targets. One hundred and twelve targets in the middle overlapping section are common targets of AD and resveratrol. (**B**) PPI network. Sizes and colors of the nodes are illustrated from big to small and blue to green in descending order of degree values.

Upon conducting a thorough database search, we successfully identified 215 targets associated with resveratrol. Furthermore, a total of 1,917 genes were retrieved from the GeneCards database, whereas 674 genes were obtained from the DisGeNET database, which are both related to AD. After eliminating redundancies, a total of 2,274 therapeutic targets were collected for this investigation. Through the utilization of a Venn diagram, we identified a total of 112 overlapping genes at the intersection of resveratrol and AD targets (Fig. **6A**).

#### 3.2.2 Protein–protein Interaction Network of AD Targets

To assess the potential implications of the identified targets in the development of complex diseases and unveil their underlying relationships, the overlapping targets were inputted into the STRING 11.0 platform to construct a protein-protein interaction network. After a comprehensive examination of the topology parameters within the protein-protein interaction network, the 112 targets were methodically ranked in descending order based on their degrees. Subsequently, these targets were organized in a concentric circle formation (Fig. **6B**). The degree of a given target reflected its significance, as determined by the number of connections formed with other nodes within the network. Using the degree parameter as a criterion, the targets located in the innermost circle were selected to predict essential therapeutic targets.

#### 3.2.3 GO and KEGG Enrichment Analysis

A comprehensive GO functional and KEGG pathway enrichment analysis was conducted to provide insights into the biological effects of resveratrol on gene functions and signaling pathways involved in treating AD. Fig. (**7**) highlights the top 10 GO items and the top 20 KEGG pathways that were selected based on the p-value.

For biological processes (BPs), the targets were mainly enriched in the positive regulation of kinase activity, the positive regulation of the MAPK cascade, and the regulation of the inflammatory response. The top 10 items primarily encompassed molecular functions such as protein kinase activity (including transmembrane receptor activity), peptidase activity, and nuclear receptor activity. The cellular components were predominantly focused on locations, such as in the membrane raft, vesicle lumen, membrane microdomain, cytoplasmic vesicle lumen, secretory granule lumen, ficolin-1-rich granule, ficolin-1-rich granule lumen, focal adhesion, cell-substrate junction, and plasma membrane raft. As per the outcomes of the KEGG pathway enrichment analysis, a significant proportion of items were associated with pathways such as the Ras signaling pathway, the PI3K-Akt signaling pathway, the Relaxin signaling pathway, the MAPK signaling pathway, the FoxO signaling pathway, the Rap1 signaling pathway, the Adherens junction, and the IL-17 signaling pathway. Notably, the common targets of resveratrol and AD exhibited significant enrichment primarily within the PI3K-Akt signaling pathway and protein kinase activity was prominently featured among the top 10 molecular function attributes.

Additionally, BPs were involved in the regulation of kinase activity and the MAPK cascade. Hence, based on our analysis, we inferred that the PI3K-Akt signaling pathway could hold a pivotal role. Furthermore, the Ras pathway and the MAPK pathway might play an important role in the regulation of AD by resveratrol.

#### 3.2.4 Component–target–pathway Network Construction

**Fig. (8):**
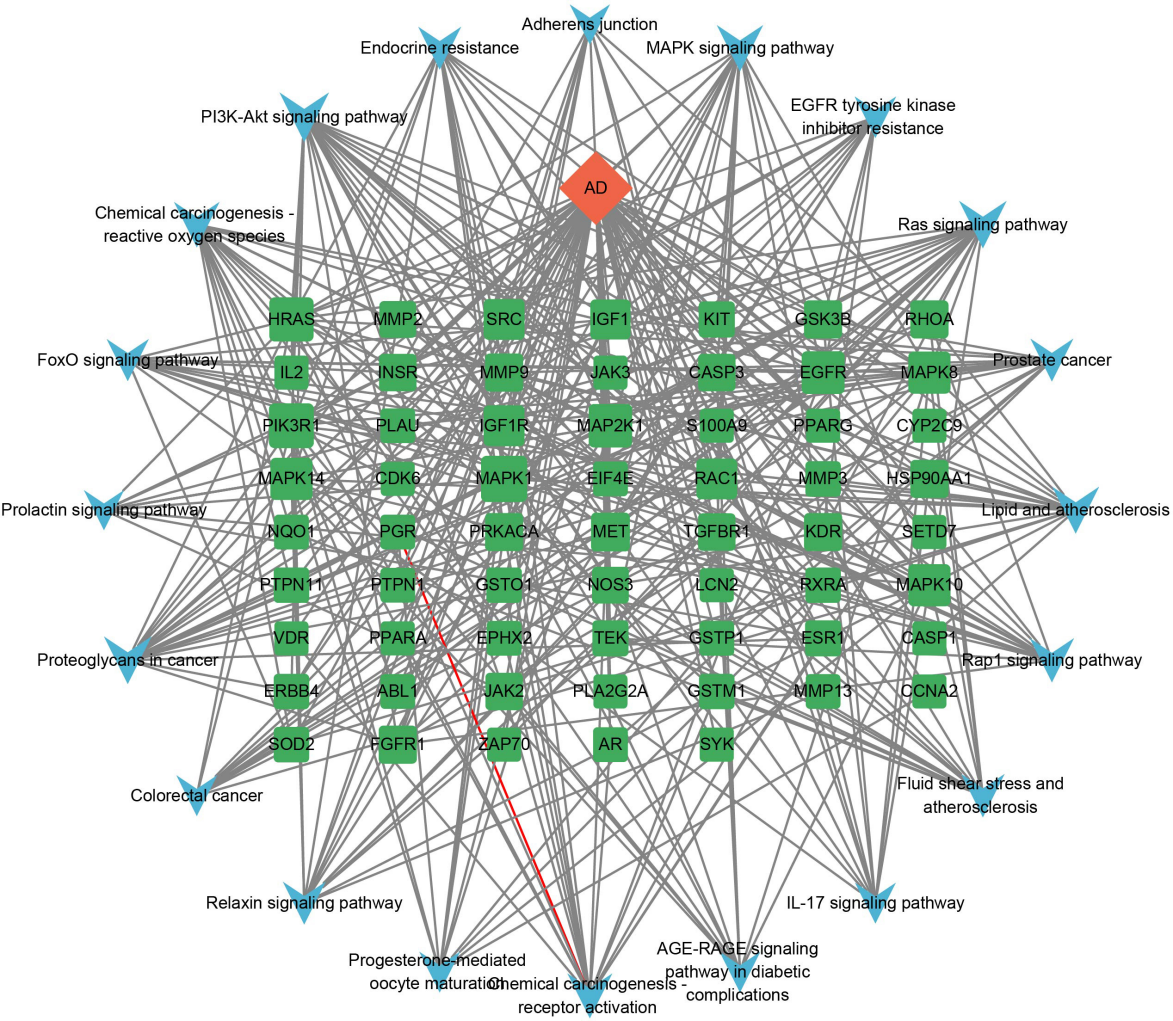
Component–target–pathway network. The orange diamond represents the bioactive component of resveratrol, 61 green squares represent targets, and 20 blue V-shapes represent pathways. Sizes of the green square node are illustrated from big to small in descending order of degree values.

Using Cytoscape, a comprehensive component-target-pathway network was established, leveraging the insights derived from the KEGG pathway enrichment analysis (Fig. **8**). Within the crucial targets identified by the protein-protein interaction network, MAPK1, HRAS, EGFR, and MAPK2K1 were notably enriched in the PI3K-Akt signaling pathway, the Ras signaling pathway, and the MAPK signaling pathway.

## 4 DISCUSSION

The aim of this meta-analysis was to evaluate the effects of resveratrol on cognitive functions, CSF and plasma markers, brain volume, and safety and tolerability in AD patients. Only five of the 2,811 scanned articles were identified for inclusion. A total of 271 patients with AD were included, with 139 in the experimental group and 132 in the placebo-treated group. The results showed that resveratrol treatment produced a significant improvement in ADAS-ADL scores (SMD=0.51; 95% CI, 0.24 to 0.78) and CSF Aβ40 (SMD=0.84; 95% CI, 0.21 to 1.47) and plasma Aβ40 levels (SMD=0.43; 95% CI, 0.07 to 0.79). However, the improvement in the resveratrol-treated group for the MMSE score, CSF Aβ42 and plasma Aβ42 levels, and brain volume was not statistically significant compared with the placebo-treated group. No significant statistical differences in the incidence of adverse effects were observed between the two groups.

Given that two of the included studies in the meta-analysis included low doses of resveratrol (5mg/day), it was possible that this may have biased the results due to differences in dose. Therefore, we recalculated all 500 mg/day dose groups and found that the results did not differ from the overall results, which may indicate that the therapeutic benefit of small doses of resveratrol for AD was still significant. In terms of cognitive function measures, although the results of our meta-analysis failed to show the significance of resveratrol in terms of MMSE scores, it was still able to show some advantages in terms of ASAD-ADL scores. Considering the number of included studies and the small sample size, our study can only go some way to support the conclusion that resveratrol can improve cognitive impairment in AD patients. Whether the therapeutic value of resveratrol can be converted into clinical applications needs to be explored in future clinical studies with larger sample sizes. Additionally, in the biomarker study we showed statistically meaningful stabilization of Aβ40 levels in both CSF and plasma in the resveratrol treatment group compared to placebo treatment. Nevertheless, the stabilizing effect of the drug on Aβ42 was not statistically significant, and even the visual comparison of Aβ42 values in CSF was not as good as that of placebo. Deposition of Aβ in the brain was a pathological hallmark of AD [48]. There were two main isoforms of Aβ: residue 42 Aβ42 and residue 40 Aβ40 [49]. The only difference between Aβ42 and Aβ40 was that Aβ42 had two additional residues at the C-terminus [50]. It was well known that amyloid plaques in the AD brain consisted mainly of Aβ42 rather than Aβ40 [51, 52], and a separate study had shown that an increase in the Aβ42/Aβ40 ratio led to an increase in the Aβ42 population alone, which contributed to the AD pathological process [53, 54]. Our study suggested that high Aβ40 actually limited Aβ42 aggregation after treatment, which suppressed neuroinflammatory activity and improved cognitive deficits.

Following network pharmacology principles, we constructed a protein-protein interaction network involving shared targets between the drug and the disease. Subsequently, we conducted GO and KEGG pathway analyses on these genes to gain deeper insights into their functions and involvement in biological processes. According to the BP enrichment, multiple genes are prominently involved in regulating kinase activity, the MAPK cascade, and the inflammatory response. These findings suggest that resveratrol may primarily act through these pathways to mitigate the progression of AD. Drawing from the insights of the KEGG pathway enrichment analysis and a review of existing literature, it was anticipated that resveratrol could potentially exert its therapeutic effects in AD predominantly through the modulation of the PI3K-Akt signaling pathway. Through analysis of the component-target-pathway network, MAPK1, HRAS, EGFR, and MAPK2K1 emerged as key targets. This suggests that the efficacy of resveratrol in treating AD is primarily associated with these identified targets.

Dysregulation of the MAPK1/MAPK2K1/HRAS/EGFR axis has been identified in AD, implicating its involvement in the pathogenesis of this neurological disorder, including neuroinflammation, tau hyperphosphorylation, and Aβ production. Based on the data acquired, multiple miRNA dysregulations have been observed to potentially modulate the MAPK signaling pathway in distinct stages of AD [55]. Furthermore, manipulating the expression of miRNAs implicated in MAPK regulation has demonstrated cognitive deficit improvement in animal models of AD [56]. Notably, miR-132 garners considerable interest owing to its distinctive neuroprotective capabilities [57], which involve the inhibition of Aβ and Tau depositions, alongside ameliorating oxidative stress, through the modulation of ERK/MAPK1 signaling. A previous study investigated the role of HRAS in AD by examining its impact on cognitive function and amyloid pathology in transgenic APP/PS1 mice [58]. The absence of HRAS rescues spatial memory deficits and reduced cortical amyloid deposition, astrogliosis, and loss of dendritic spines associated with amyloid plaques in these mice. These findings suggest that HRAS plays a crucial role in AD pathogenesis and could be a potential therapeutic target. EGFR plays a crucial role in early brain development, but its expression decreases as the nervous system matures. However, in instances of neural function decline and brain atrophy, EGFR expression reoccurs to maintain neuronal homeostasis [59]. In AD, the neurotoxic amyloid beta fragment (Aβ1-42) enhances EGFR expression, leading to sustained phosphorylation of downstream signaling pathways, subsequent extensive Aβ1-42 production, and tau phosphorylation, contributing to the progression of the disease [59, 60]. Therefore, it suggesting that resveratrol can exhibit potential in modulating the trajectory of AD by targeting these identified key factors.

The PI3K signaling pathway plays an important role in the treatment of AD with resveratrol [61]. One of the key mechanisms by which resveratrol modulates the PI3K-AKT pathway is through the activation of the upstream receptor tyrosine kinases, such as EGFR. Activation of these receptors initiates a cascade of intracellular events, ultimately leading to the activation of PI3K and subsequent phosphorylation of AKT. Resveratrol has been shown to enhance the activation of EGFR, thereby promoting the activation of the PI3K-Akt pathway in neuronal cells [62]. Furthermore, resveratrol has been found to directly inhibit the activity of PTEN, a negative regulator of the PI3K-Akt pathway [63]. PTEN acts by dephosphorylating PIP3, the product of PI3K, thus inhibiting Akt activation [64]. By inhibiting PTEN, resveratrol increases the levels of phosphorylated Akt, leading to enhanced downstream signaling and neuroprotective effects [65]. Moreover, resveratrol has been reported to modulate other components of the PI3K-Akt pathway, such as mTOR [66] and GSK3β [67]. Activation of mTOR and inhibition of GSK3β are key downstream events of the PI3K-Akt pathway and play important roles in neuronal survival and synaptic plasticity [68].

Although our findings demonstrate the potential benefits of resveratrol in AD, it is important to acknowledge the limitations of our analysis. Firstly, the evaluation of cognitive function outcomes is inadequately represented, primarily due to the limited number of relevant clinical trials available. Secondly, different doses of resveratrol may introduce bias and clinical heterogeneity into our analyses. Therefore, we performed random effects models and subgroup analyses, which are better for studies with potential heterogeneity [69, 70]. Furthermore, although we have identified the pertinent targets and pathways through which resveratrol may act in AD treatment, further experimental research is necessary to validate our hypothesis. We hope that a sufficient number of forthcoming studies will substantiate and corroborate our supposition and validate the envisioned molecular mechanism.

## CONCLUSION

Through a comprehensive approach involving meta-analysis and network pharmacology, our research systematically unveiled the intricate molecular mechanisms through which resveratrol exerts its influence on AD. From intricate networks, we forecasted four pivotal targets, leading us to the conclusion that the therapeutic impact of resveratrol on AD involves the inhibition of the PI3K signaling pathway. We anticipate that this study will offer supplementary guidance regarding the potential medicinal role of resveratrol in AD treatment. The findings present avenues for further investigation in the future, building upon the insights gained from this research.

## Figures and Tables

**Fig. (1):**
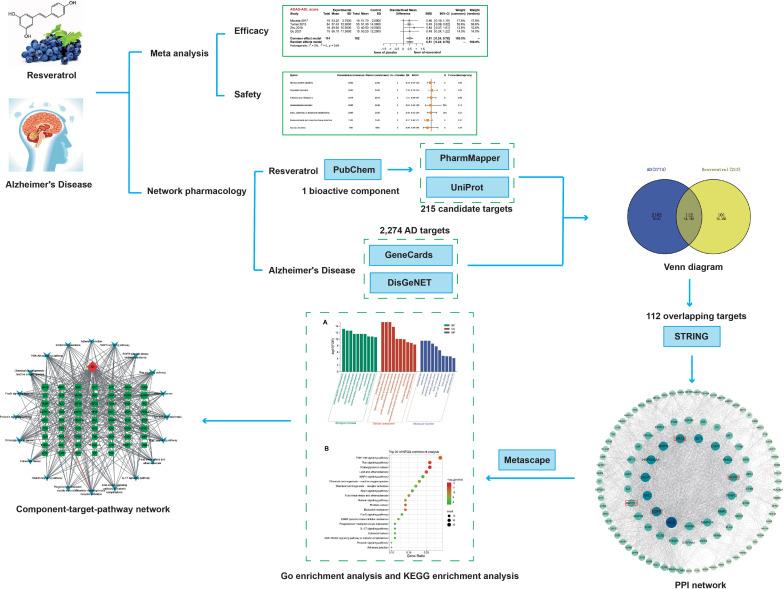
The flow chart for meta-analysis and network pharmacology.

**Fig. (5):**
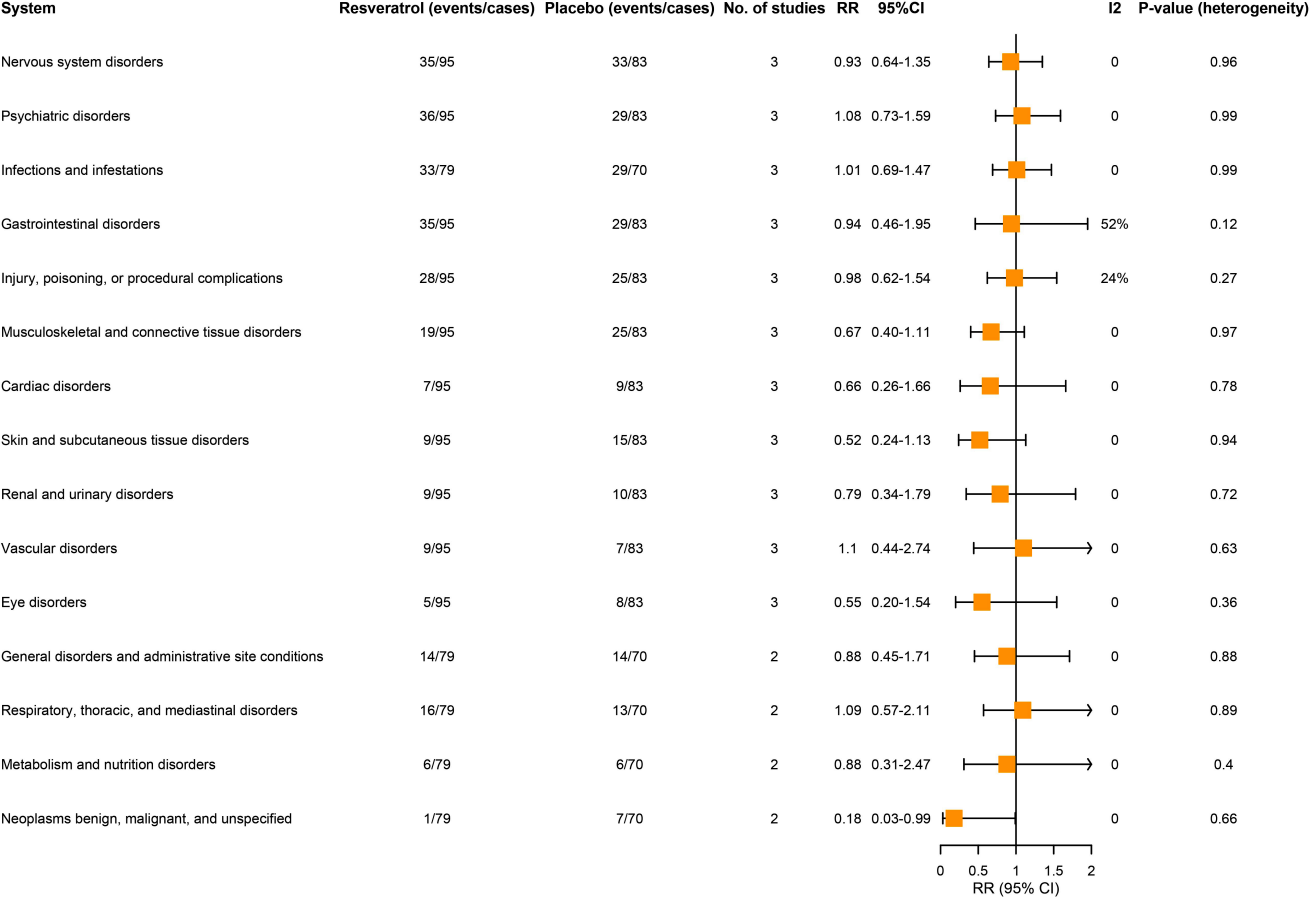
Meta analysis of the safety of resveratrol *versus* placebo in the treatment of AD patients.

**Fig. (7):**
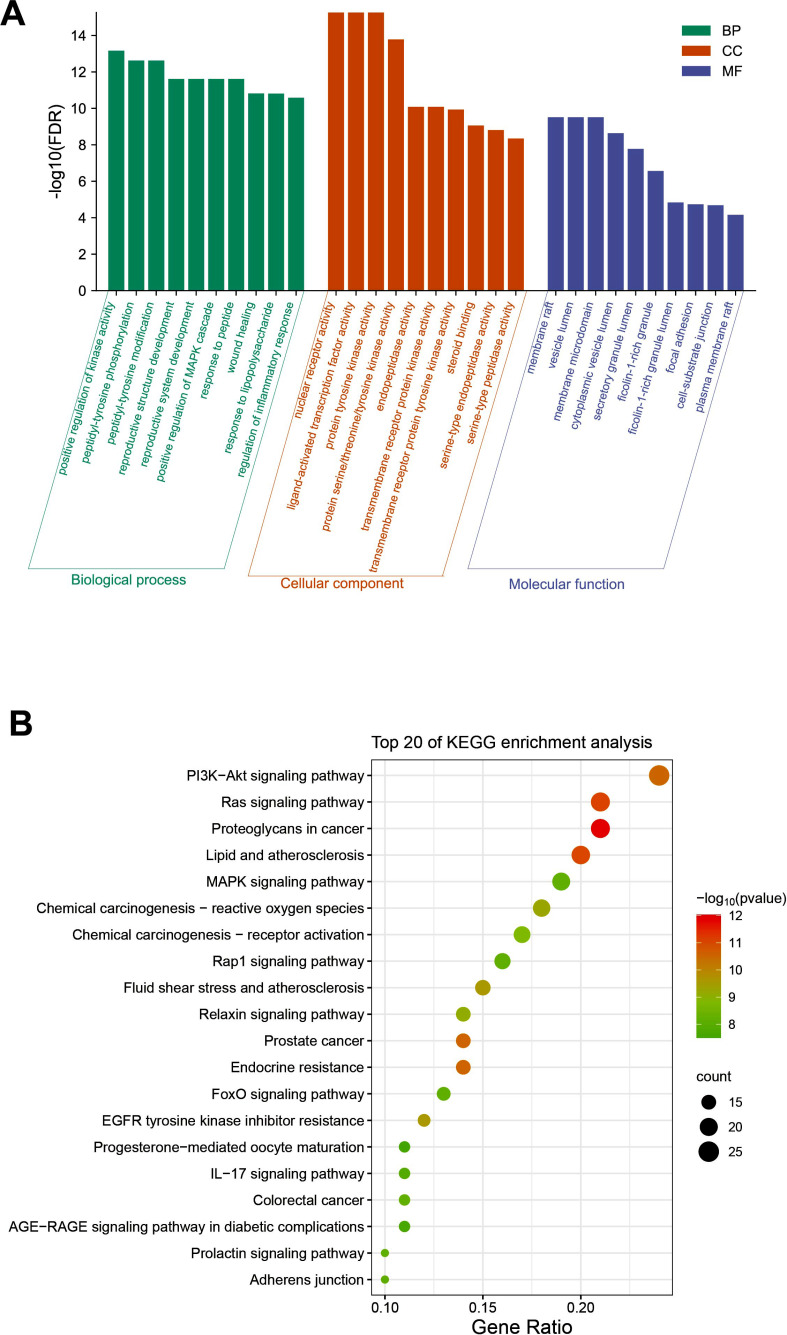
Enrichment analysis of the targets of resveratrol in treating AD. (**A**) GO functional analysis. Top 10 items of each part are shown. (**B**) KEGG pathway enrichment analysis. The sizes of the bubbles are illustrated from big to small in descending order of the number of potential targets involved in the pathways.

**Table 1:** Characteristics of included studies.

**Study**	**Year**	**Country**	**Characteristic (Intervention *vs*. Placebo)**	**No. of ** **Patients**	**Age (years; mean ± SD)**	**Female (N, %)**	**Population Baseline Characteristics**	**Diagnosis ** **Criteria** **for AD**	**Follow-up** **(weeks)**
Blass	2006	USA	RGM (resveratrol: 5mg, glucose: 5g, malate: 5g) administered twice daily in 15 mL liquid form, dissolved in commercial grape juice	ADAS-cog: 20 MMSE: 25	74.0 ± 2.0	NA (47%)	ADAS-cog score: 22.0 ± 3.0 MMSE score: 20.0 ± 1.0	A complete medical and neurological history and physical examination, brain imaging, and clinical laboratory studies	12
			Composed of sucralose and lemon juice and indistinguishable by color or flavor from the active preparation	ADAS-cog: 29 MMSE: 30	74.0 ± 1.0	NA (46%)	ADAS-cog score: 23.0 ± 2.0 MMSE score: 19.0 ± 0.8		
Moussa	2017	USA	Resveratrol 500 mg orally once daily (with a dose increase in increments of 500 mg every 13 weeks, ending with 1000 mg twice daily)	19	NA	NA	ADAS-ADL score: 61.2 ± 3.3 MMSE score: 19.3 ± 1.3	NINCDS/ADRDA	52
			The type of placebo used was not reported, but an identical placebo was provided, in accordance with current GMP guidelines	19	NA	NA	ADAS-cog score: 65.0 ± 1.7 MMSE score: 19.4 ± 1.0		
Turner	2015	USA	Resveratrol 500 mg orally once daily (with a dose increase in increments of 500 mg every 13 weeks, ending with 1000 mg twice daily)	64	69.8 ± 7.7	40 (62.5)	ADAS-cog score: 25.3 ± 10.1 ADAS-ADL score: 63.7 ± 10.8 MMSE score: 20.2 ± 4.4 NPI score: 7.5 ± 7.9	NINCDS/ADRDA	52
			An identical placebo was provided, in accordance with current GMP guidelines	55	73.0 ± 8.2	28 (50.9)	ADAS-cog score: 23.7 ± 8.6 ADAS-ADL score: 60.5 ± 10.7 MMSE score: 20.7 ± 4.3 NPI score: 11.1 ± 11.6		
Zhu	2018	USA	RGM (resveratrol: 5mg, glucose: 5g, malate: 5g) administered twice daily in 15 mL liquid form, dissolved in commercial grape juice	16	80.5 ± 8.6	5 (31.3)	ADAS-cog score: 26.4 ± 11.9 ADAS-ADL score: 49.1 ± 10.3 MMSE score: 18.1 ± 4.9 NPI score: 5.4 ± 5.9	NINCDS/ADRDA	52
			Composed of sucralose and lemon juice and indistinguishable by color or flavor from the active preparation	13	79.3 ± 6.5	5 (38.5)	ADAS-cog score: 29.2 ± 8.9 ADAS-ADL score: 46.6 ± 7.6 MMSE score: 19.4 ± 3.8 NPI score: 7.9 ± 11.8		
Gu	2021	China	Trans-resveratrol 500 mg orally once daily	15	71.5 ± 6.5	7 (46.6)	ADAS-cog score: 24.3 ± 9.5 ADAS-ADL score: 61.3 ± 13.2 MMSE score: 20.3 ± 3.2 NPI score: 9.8 ± 6.5	NINCDS/ADRDA	52
			An identical placebo was provided in accordance with current GMP guidelines	15	72.8 ± 5.3	7 (46.6)	ADAS-cog score: 25.1 ± 10.3 ADAS-ADL score: 60.9 ± 12.4 MMSE score: 20.4 ± 2.9 NPI score: 9.5 ± 7.7		

**Annotation:** USA, the United States of America; RGM, resveratrol/glucose/malate; ADAS-cog, Alzheimer's Disease Assessment Scale—Cognitive Subscale; ADAS-ADL, Alzheimer's Disease Cooperative Study—Activities of Daily Living; MMSE, Mini-Mental State Examination; AD, Alzheimer's Disease; NA, not available.

## Data Availability

The datasets used and/or analyzed during the current study are available from the corresponding author upon reasonable request.

## LIST OF ABBREVIATIONS

AD: Alzheimer’s Disease
SMD: Standardized Mean Difference
CI: Confidence Interval
ADAS-ADL: Alzheimer’s Disease Cooperative Study-Activities of Daily Living
CSF: Cerebrospinal fl-uid
MMSE: Mini-Mental State Examination
Aβ: Amyloid-beta
ADAS-cog: Alzheimer’s Disease Assessment Scale-Cognitive Subscale
ROB: Risk of bi-as
GO: Gene ontol-ogy
KEGG: Kyoto Encyclopedia of Genes and Genomes
BPs: Biological pro-cesses

## SUPPLEMENTARY MATERIAL

PRISMA checklist is available on the publisher’s website Supplementary material is available as supplementary material on the publisher’s website along with the published article.
