# Supplementary material for: Evidence of Clinical Efficacy and Pharmacological Mechanisms of Resveratrol in the Treatment of Alzheimer’s Disease
Source: Curr Alzheimer Res. 2023 Dec 30;20(8):588–602. doi: 10.2174/0115672050272577231120060909 (PMC10825797; doi:10.2174/0115672050272577231120060909)
Supplement: Supplementary file 1 — PRISMA checklist is available on the publisher’s website Supplementary material is available as supplementary material on the publisher’s website along with the published article. [file CAR-20-588_SD1.pdf]

Supplementary Materials

Evidence of Clinical Efficacy and Pharmacological Mechanisms of Resveratrol in the Treatment of Alzheimer's Disease

Sian Jin[1,#], Xuefeng Guan[2,*] and Dongyu Min[3,*]

[1]Liaoning University of Traditional Chinese Medicine, Shenyang, 110000, China; [2]Shenyang Pharmaceutical University, Shenyang, 110000, China; [3]Liaoning Hospital of TCM, Shenyang, 110000, China

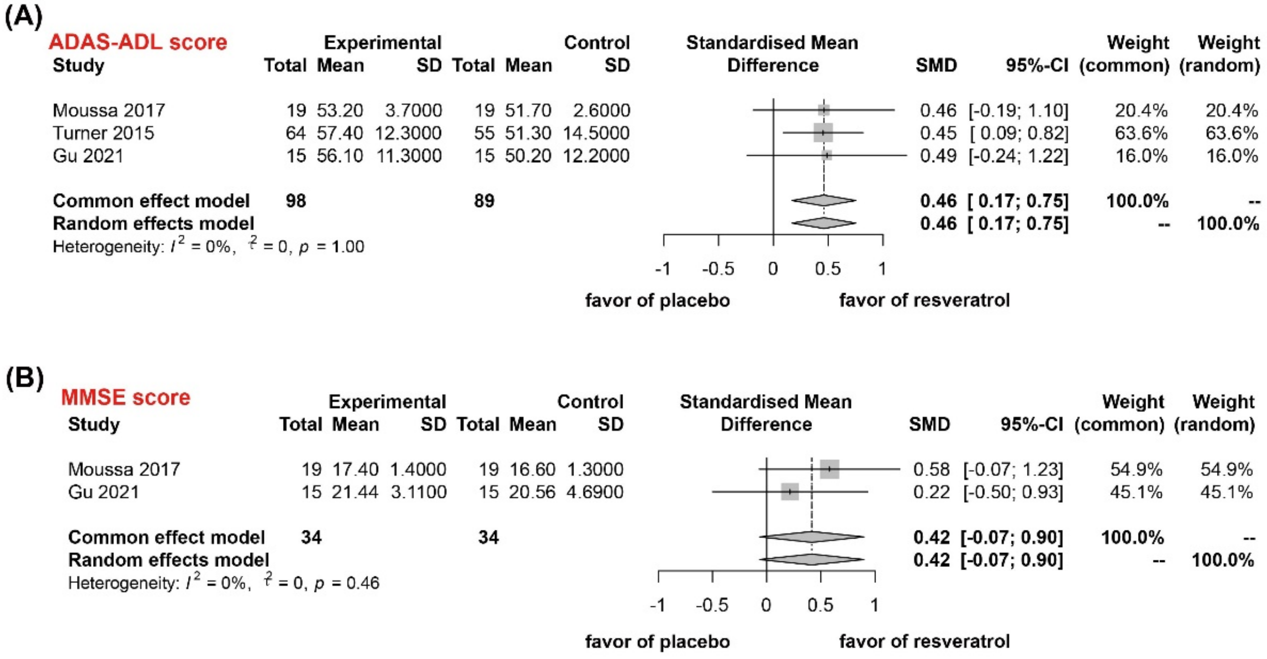

Fig. (S1). Subgroup analysis of resveratrol in the treatment of AD in terms of ADAS-ADL score **(A)** and MMSE score **(B)**.

 © 2023 Bentham Science Publishers
